# An Investigation of the Energy-Absorption Characteristics of Thin-Walled Polymer Composite C-Channels: Experiment and Stacked Shell Simulation

**DOI:** 10.3390/polym16152099

**Published:** 2024-07-23

**Authors:** Xiaomin Zhang, Haolei Mou, Shanshan Song, Zhenyu Feng

**Affiliations:** 1 College of Safety Science and Engineering, Civil Aviation University of China, Tianjin 300300, China; xm-zhang@cauc.edu.cn; 2 Engineering Techniques Training Center, Civil Aviation University of China, Tianjin 300300, China; 3 Science and Technology Innovation Research Institute, Civil Aviation University of China, Tianjin 300300, China; 4 Sino-European Institute of Aviation Engineering, Civil Aviation University of China, Tianjin 300300, China

**Keywords:** thin-walled polymer composite, axial collapse, failure mechanism, energy absorption, finite-element model

## Abstract

Polymer composite materials are increasingly used in civil aircraft structures. The failure mode and energy-absorption characteristics of polymer composite structures have garnered significant attention from academia and industry. For thin-walled polymer composite C-channels with layups of [0/90]_3s_, [45/-45]_3s_, and [45/90/-45/0]_3_, low-speed axial compression tests were performed to investigate the failure modes, failure mechanisms, and energy-absorbing characteristics. After parametric studies using [0] and [90] single-element models, stacked shell models of thin-walled composite C-channels were established using the Lavadèze single-layer damage constitutive model, Puck 2000, and Yamada Sun failure criteria. The results show that these thin-walled composite C-channels exhibit a stable progressive crushing process with a local buckling failure mode, encompassing local buckling, fiber break-age, matrix cracks, delamination, and corner cracking. The stacked shell model demonstrates reasonable agreement with the progressive crushing process of thin-walled composites, accurately capturing interlayer matrix failure and interface delamination cracking behavior. A comparison of the specific energy absorption (*SEA*) and mean crushing force (*F*_mean_) between the simulation and test results yields a difference of less than 6%, indicating a strong correlation between the simulation results and the experimental energy-absorbing characteristics. It also shows that a deep understanding of the parameters is helpful for accurate numerical modeling.

## 1. Introduction

Due to the advantages of low density, high specific strength, high specific modulus, and great potential for the integrated design of structural materials, polymer composite structures are increasingly used in the automobile and aerospace industries [1,2,3,4]. In recent years, polymer composite structures have played a major role in the primary load-bearing structures and energy-absorbing structures of large aircraft. For composite fuselage structures, an equivalent level of crashworthiness must be provided compared to similar traditional metal fuselage structures under emergency landing conditions [5,6], which mainly ensures that the acceleration and loads experienced by occupants during impact events are consistent with those specified in FAR/CCAR 25.562 (b) and provide sufficient living space for occupants.

The most effective and economical means to meet the crashworthiness requirements is to arrange thin-walled polymer composite structures in the fuselage section to absorb large amounts of impact kinetic energy in a controlled manner [7,8,9]. Taking the Boeing 787 [10], a series of thin-walled polymer composite C-channels were installed under the cargo subfloor, affecting the failure modes and crashworthiness of the fuselage section. Therefore, much research has been carried out by the Boeing Company, the Federal Aviation Administration, and others to verify and improve crashworthiness, achieving great success [11,12]. In recent decades, various thin-walled polymer composite structures with different shapes have been put forward and their axial collapse behaviors studied, including circular tubes [13,14,15], square tubes [16,17], corrugated plates [18,19,20], conical shells [21], composite-reinforced aluminum tubes [22,23], and filled structures [24,25]. Factors such as the trigger [25,26,27], geometric features [28,29,30], mechanical properties of the fiber and matrix [31,32], and layup [33] have also been studied.

With the development of finite-element codes, such as PAM-CRASH [34], LS-DYNA [35], and ABAQUS [36], the combination method, i.e., many finite-element simulations with a small number of experiments, has become an effective way to study the energy-absorbing characteristics of composite structures and to design composite energy-absorbing structures [37,38,39]. Researchers have carried out extensive numerical simulation work, and various failure criteria are employed to predict material failure under different loading conditions. The most commonly used failure criteria include the maximum stress criterion [40,41], maximum strain criterion [42], Tsai–Hill criterion [43], Hashin criterion [44], Puck’s failure criterion [45], LaRC criterion [46], and Yamada–Sun criterion [47]. In addition, the finite-element modeling of composite thin-wall tubes has been researched using single-layer shell models and multi-layer shell models. Palanivelu et al. [48] developed single-layer and two-layer shell models of glass fiber composite thin-wall cylindrical and square tubes, respectively. In the two-layer model, solid adhesive units were established between the two shell elements, and predefined cracks were set in the shell elements to simulate crack propagation. The results show that the single-layer shell model could not capture delamination failure accurately, leading to inaccurate results, whereas the multi-layer shell model could simulate the failure morphology and energy-absorbing characteristics consistent with the test results. Siromani et al. [49] developed the multi-layer shell modeling method to numerically simulate the crushing process of composite cylindrical tubes using the LS-DYNA MAT 54 material model, achieving relatively accurate simulation of the chamfered end and boundary conditions. The average compressive load and *SEA* obtained by the simulation were relatively in accordance with the test results, though the initial peak load was 8–20% higher. Johnson et al. [50] numerically simulated the quasi-static crush test of [0/90]_8_ thin-walled structures using the stacked shell model. A rigid element was built at the top of the model, and offset nodes were situated in the upper part of the two shell elements that were located in the middle. In this way, the interlayer separation during the initial compression phase can be simulated. The simulated average compressive load was similar to the experimental results, which verified the effectiveness of the modeling method.

In addition, Feraboli et al. [51,52] explored the LS-DYNA MAT 54 material model and the Chang–Chang failure criteria [53,54], developed by Chang and Chang, which distinguish between tension and compression failures in the fiber and matrix of composite materials. The criteria include fiber tensile failure, fiber compressive failure, matrix tensile failure, matrix compressive failure, and shear failure. The key parameters of the constitutive relations, damage evolution, and failure criteria were identified, and their sensitivity was analyzed. The results exhibit that some model parameters, which are either non-physical or cannot be measured experimentally, enormously influence the simulation. It is expected to lack predictive capability before parameter calibration. However, the study was confined to LS-dyna MAT54, and similar research on other material models is still rarely seen.

In the present work, three groups of thin-walled polymer composite C-channels with different layups, i.e., [0/90]_3s_, [45/-45]_3s_, and [45/90/-45/0]_3_, were analyzed for failure modes, failure mechanisms, and energy-absorbing characteristics by conducting low-speed axial compression tests. The [0] and [90] single-element Lavadèze models were developed to conduct parametric analysis, investigate the sensitivity of various input parameters, and determine the most appropriate parameters based on the stress–strain relationship. Subsequently, the stacked shell models of thin-walled polymer composite C-channels were established using the Lavadèze single-layer damage constitutive model. Owing to the advantages in matrix failure and transverse tensile and shear stress interaction evaluation, the Puck 2000 failure criteria and Yamada–Sun failure criteria have been chosen. These models are further verified based on test results and energy-absorbing characteristics. These findings enhanced the understanding of the behavior of composite materials under axial compression and provided a strategy for calibrating finite-element models.

## 2. Specimens and Experimental

### 2.1. Specimens Design and Manufacture

**Figure 1:**
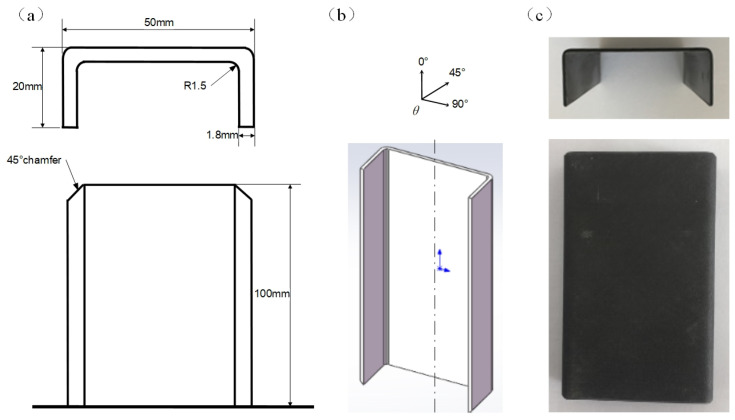
Schematic diagram of the polymer composite C-channels (**a**), three-dimensional stereogram (**b**), and C-channel specimen (**c**).

In the sub-cargo of aircraft, thin-walled polymer composite C-channels were designed to support the cargo floor crossbeam. During a crash event, they can transfer and absorb the impact load. Thin-walled polymer composite C-channels were fabricated using the T700/MTM28 carbon fiber reinforced epoxy composite provided by AVIC Manufacturing Technology Institute (Beijing, China) through a hot-pressing process. The fiber volume content was approximately 60% according to the standard method in DIN EN ISO 7822 [55]. T700 carbon fibers provide a high specific strength and modulus, making them ideal for aerospace applications where weight reduction and mechanical performance are critical; furthermore, extensive research has validated the effectiveness of T700/MTM28 composites in energy absorption and crashworthiness [18,19,51], making them a well-understood and reliable choice for further study. The geometric configuration of the C-channels is as shown in Figure 1. The total height of the C-channels is 100 mm, with a width of 50 mm. The left and right flanges measure 20 mm in width. The transition arc between flat segment and flange has an inner radius of 1.5 mm, while the outer radius is equivalent to 1.5 mm plus the thickness of the C-channel. A 45° outer chamfer was milled at one end of the C-channel, as shown in Figure 1a. θ is the fiber angle, as shown in Figure 1b. The fiber direction is consistent with the axial direction of the C-channel, if θ = 0°. The thin-walled polymer composite C-channel specimens, as shown in Figure 1c, were designed with a thickness of 1.8 mm, corresponding to 12 layers, i.e., [0/90]_3s_, [45/-45]_3s_, and [45/90/-45/0]_3_. Each type of C-channel was tested at least 3 times to ensure the consistency and reliability of the results.

### 2.2. Experimental Procedures

**Figure 2:**
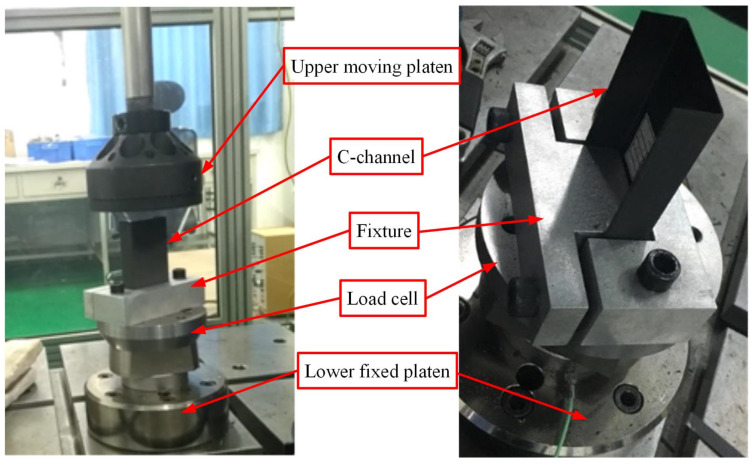
Test scheme, fixture and C-channel.

Low-speed axial compression tests of thin-walled composite C-channels were conducted at room temperature using the INSTRON VHS 160/100-20 (Norwood, MA, USA). The 45° chamfer of the thin-walled composite C-channel was set at the top and free, while the other end was fixed using a clamp mounted on the plate by two bolts. A load cell was installed between the plate and the lower fixed platen to capture the load–time history curve, as shown in Figure 2. The upper moving platen moved downward at a constant velocity of 50 mm/s. The entire process of the low-speed axial compression tests was recorded using PHOTRAN high-speed video (Tokyo, Japan) at 2000 frames per second. Following the experiments, the C-channels were scanned using the ZEISS METROTOM (Deutschland, GmbH, Düsseldorf, Germany) industrial computed tomography (CT) scanning machine.

### 2.3. Energy-Absorbing Metrics

(1) Energy absorption (*EA*, unit: kJ): The total energy absorbed by the integration of the crushing force over the entire crushing distance.

(1) EA=∫Fdl

where *F* is the crushing force.

(2) Specific energy absorption (*SEA*, unit: kJ/kg): The energy absorbed per unit of mass of the structure. The *SEA* is an important parameter to measure the energy-absorbing capabilities. The ratio of the total energy to the mass is the *SEA*, which can be calculated as follows:

(2) $$SEA=\frac{EA}{\rho AL}$$

where ρ is the density, *A* is the effective cross-sectional area, and *L* is the total crushing distance.

(3) Peak crushing force (*F_max_*, unit: kN): The threshold value of structural damage, which is used to evaluate the structural failure under an external force. This parameter is the peak crushing force of the load–displacement curve.

(4) Mean crushing force (*F_mean_*, unit: kN): The mean crushing force during the entire crushing process, which can be calculated as follows:

(3) $$F_{mean}=\frac{EA}{L}$$

## 3. Numerical Study

### 3.1. Constitutive Model

The Lavadèze orthotropic single-layer composite model was used, which judges the matrix damage by using the Puck inter fiber failure (IFF) criteria combined with the Yamada–Sun fiber failure criteria. The failure criteria of the combination of the fiber and matrix can take the failure of the fiber under tensile and compressive loads, the formation of matrix microcracks under a transverse tensile load, and the debonding of the fiber and the matrix under the shear load into consideration. The constitutive relation of the Lavadèze single-layer composite model is shown in Equation (4):

(4) $$\left\{\begin{array}{l} \varepsilon_{11}^{e}\\ \varepsilon_{22}^{e}\\ 2\varepsilon_{12}^{e}\\ 2\varepsilon_{23}^{e}\\ 2\varepsilon_{13}^{e} \end{array}\right\}=\left[\begin{array}{ccccc} \frac{1}{E_{1}} & -\frac{\nu_{12}^{0}}{E_{1}} & 0 & 0 & 0\\ -\frac{\nu_{12}^{0}}{E_{1}} & \frac{1}{E_{2}} & 0 & 0 & 0\\ 0 & 0 & \frac{1}{G_{12}} & 0 & 0\\ 0 & 0 & 0 & \frac{1}{G_{23}^{0}} & 0\\ 0 & 0 & 0 & 0 & \frac{1}{G_{13}} \end{array}\right]\left\{\begin{array}{l} \sigma_{11}\\ \sigma_{22}\\ \sigma_{12}\\ \sigma_{23}\\ \sigma_{13} \end{array}\right\}$$

where Direction 1 of the natural coordinates is the fiber direction; Direction 2 is the vertical fiber direction; Direction 3 is the normal direction of the unidirectional composite layer; *ε*_11_ is the strain in the direction of the fiber; *ε*_22_ is the strain in the vertical direction of the fiber; *ε*_12_, *ε*_23_, and *ε*_13_ are the shear strains in the corresponding directions; *σ*_11_ is the stress in the direction of the fiber; *σ*_22_ is the stress in the vertical direction of the fiber; *σ*_12_, *σ*_23_, and *σ*_13_ are the shear stresses in the corresponding directions; *E*_1_ and *E*_2_ are the elastic moduli in the direction of the fiber and the vertical fiber direction; *G*_12_ is the shear modulus of the plane 1, 2; *G*_23_ is the shear modulus of the plane 2, 3; *G*_13_ is the shear modulus of the plane 1, 3; and *ν*_12_ is the Poisson’s ratio.

### 3.2. Failure Criteria

The Puck’s IFF criterion in natural coordinates is shown in Equation (5):

(5) $$\lambda^{M}=\left\{\begin{array}{l} \sqrt{{\left(\frac{\sigma_{12}}{R_{12}}\right)}^{2}+{\left(1-P_{12}^{+}\frac{R_{22}^{+}}{R_{12}}\right)}^{2}\left(\frac{\sigma_{22}}{R_{22}^{+}}\right)}+P_{12}^{+}\frac{\sigma_{22}}{R_{12}}(\sigma_{22}\geq 0)\\ \frac{1}{R_{12}}\left(\sqrt{{\left(\sigma_{12}\right)}^{2}+{\left(p_{12}^{-}\sigma_{22}\right)}^{2}}+p_{12}^{-}\sigma_{22}\right)(\sigma_{22}<0,\;0\leq \left|\frac{\sigma_{22}}{\sigma_{12}}\right|\leq \frac{R_{22}^{A}}{\left|\sigma_{12}^{c}\right|})\\ \left[{\left(\frac{\sigma_{12}}{2\left(1+p_{22}^{-}\right)R_{12}}\right)}^{2}+\left(\frac{\sigma_{22}}{R_{22}^{-}}\right)\right]\frac{R_{22}^{-}}{\left(-\sigma_{22}\right)}(\sigma_{22}<0,\;0\leq \left|\frac{\sigma_{12}}{\sigma_{22}}\right|\leq \frac{\left|\sigma_{12}^{c}\right|}{R_{22}^{A}}) \end{array}\right.$$

where *λ^M^* is the matrix damage judgment factor; when *λ^M^* exceeds 1, matrix failure will occur; $R_{22}^{+}$, $R_{22}^{-}$, and $R_{12}$ represent, respectively, the transverse tensile strength, transverse compressive strength, and matrix shear strength; $p_{12}^{+}$ and $p_{12}^{-}$ are matching parameters of the curve slope, which need to be determined by a multi-axis test.

The Yamada–Sun fiber failure criteria are shown in Equation (6):

(6) $$\begin{array}{l} Fail=\sqrt{{\left(\frac{\varepsilon_{fib}}{\varepsilon_{11}^{+}}\right)}^{2}+{\left(\frac{\varepsilon_{12}}{\varepsilon_{12}^{+}}\right)}^{2}+{\left(\frac{\varepsilon_{13}}{\varepsilon_{13}^{+}}\right)}^{2}},\varepsilon_{fib}>0\\ Fail=\sqrt{{\left(\frac{\varepsilon_{fib}}{\varepsilon_{11}^{-}}\right)}^{2}+{\left(\frac{\varepsilon_{12}}{\varepsilon_{12}^{-}}\right)}^{2}+{\left(\frac{\varepsilon_{13}}{\varepsilon_{13}^{-}}\right)}^{2}},\varepsilon_{fib}<0\\ \varepsilon_{fib}=\varepsilon_{11}+\nu_{12}(1-d)\varepsilon_{22} \end{array}$$

where $\varepsilon_{11}^{+}$, $\varepsilon_{12}^{+}$, and $\varepsilon_{13}^{+}$ represent the fiber tensile failure strain, positive in-plane shear failure strain, and positive out-of-plane shear failure strain, respectively; $\varepsilon_{11}^{-}$, $\varepsilon_{12}^{-}$, and $\varepsilon_{13}^{-}$ represent the fiber compression failure strain, negative in-plane shear failure strain, and negative out-of-plane shear failure strain, respectively.

### 3.3. Parametric Analysis

To characterize the basic elasticity, initial failure, and post-failure behavior of a single-element model, the [0] and [90] single-element models are established to obtain the stress–strain curves, and the influence of each parameter on the material performance is determined. Combined with the performance parameters of T700/MTM28, the set values of the material model parameters are determined by inverting the relevant parameters of the material model, which can provide the basis values of the input parameters for the finite-element modeling of thin-walled composite C-channels. A summary of the parametric studies performed is shown in Table 1.

#### 3.3.1. [0] Single-Element Model

**Figure 3:**
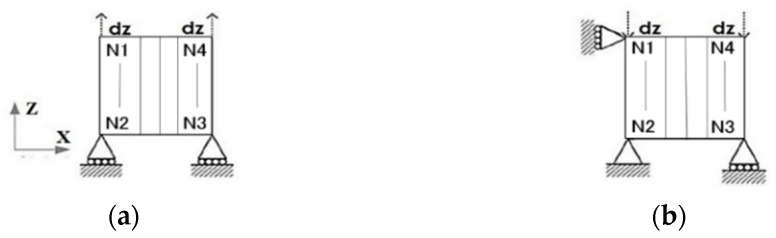
Single-element shell models with tension load (**a**) and compression load (**b**).

A square single-element model of a Belytschko–Tsay shell is developed, with a side length of 1 mm and a thickness of 0.15 mm. The tension/compression load and boundary conditions are shown in Figure 3, and the stress–strain curve can be extracted.

**Figure 4:**
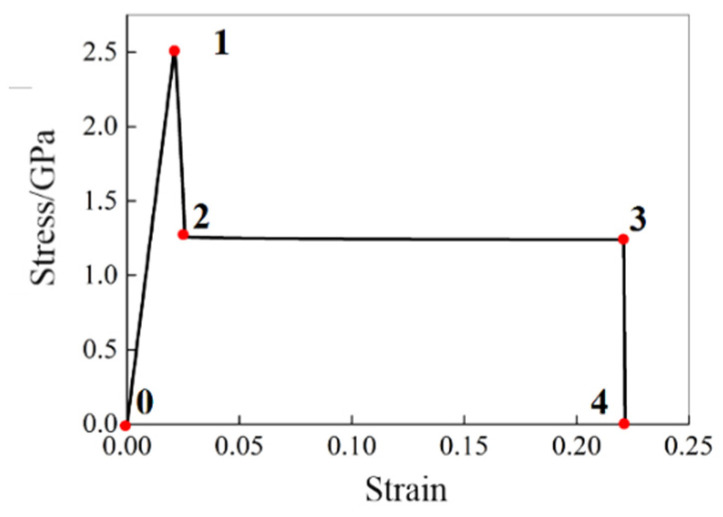
Typical stress–strain curve of the [0] single-element model, point 0 represents the initial state of the material before any load is applied, point 1 indicates the yield point of the material, point 2 represents the stress value after material degradation, point 3 represents the end of the platform stage, and point 4 represents the element deletion.

For the [0] single-element model, the typical stress–strain curve, shown in Figure 4, can be divided into the following four stages: the linear elastic stage (0–1), degradation stage (1–2), platform stage (2–3), and element deleting stage (3–4).

**Figure 5:**
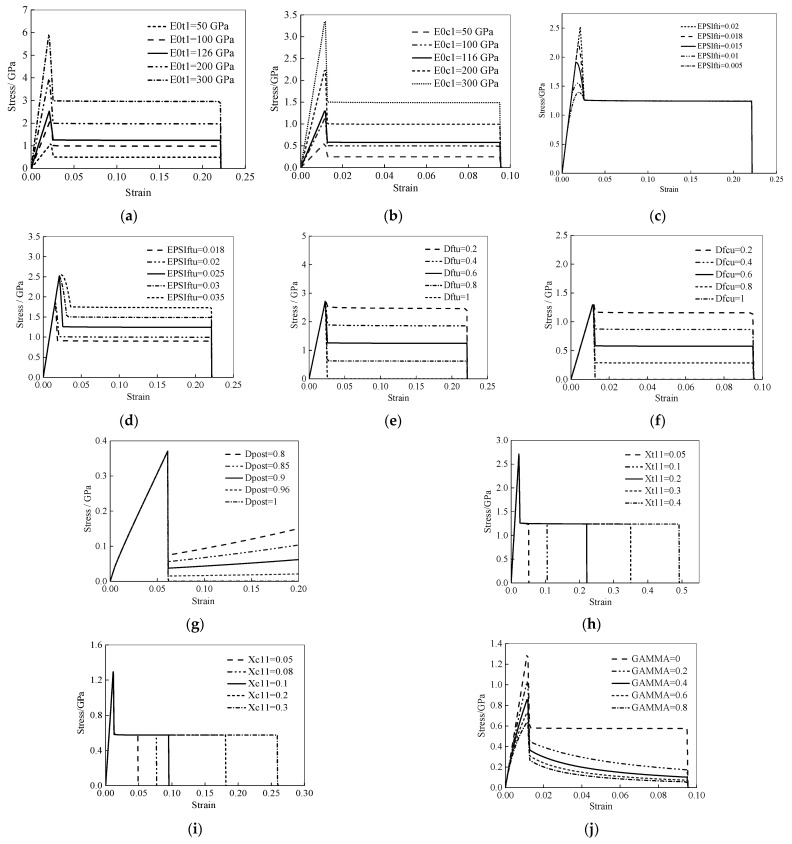
Stress–strain curves of the [0] single-element model, E0t1 (**a**), E0c1 (**b**), EPSIfti (**c**), EPSIftu (**d**), Dftu (**e**), Dfcu (**f**), Dpost (**g**), Xt11 (**h**), Xc11 (**i**), and GAMMA (**j**).

The effects of E0t1 and E0c1 on the stress–strain curve are similar, which can determine the slope of the linear elastic stage (0–1) in the stress–strain curve during the tension and compression processes, respectively, as shown in Figure 5a,b. The EPSIfti can determine the maximum strain value of the linear elastic stage (0–1) in the stress–strain curves, as shown in Figure 5c. The EPSIftu can determine the strain value (Point 2 in Figure 4 at the junction between the degradation stage (1–2) and the platform stage (2–3). The greater the EPSIftu, the larger the strain value and the greater the strength after failure, as shown in Figure 5d.

The effects of Dftu and Dfcu on the stress–strain curve are similar, which can determine the strength value (Point 3 in Figure 4) of the platform stage (2–3) in the stress–strain curve during the tension and compression processes, respectively, as shown in Figure 5e,f. The greater the Dftu and Dfcu, the smaller the strength after degradation.

The Dpost can mainly affect the strength of the matrix after degradation. The smaller the Dpost, the greater the strength after the degradation of the matrix, as shown in Figure 5g.

The Xt11 and Xc11 can determine the strain value (Point 4 in Figure 4) of the element deleting stage (3–4) in the stress–strain curve, as shown in Figure 5h,i. The greater the Xt11 and Xc11, the later the element is deleted. 

The GAMMA can affect the compressive elastic modulus and further affect the strength limit and the post-degradation compressive strength, as shown in Figure 5j. The value range of GAMMA is [0, 1], which can adjust the linearity of the compressive modulus before failure and the corresponding strength level.

#### 3.3.2. [90] Single-Element Model

**Figure 6:**
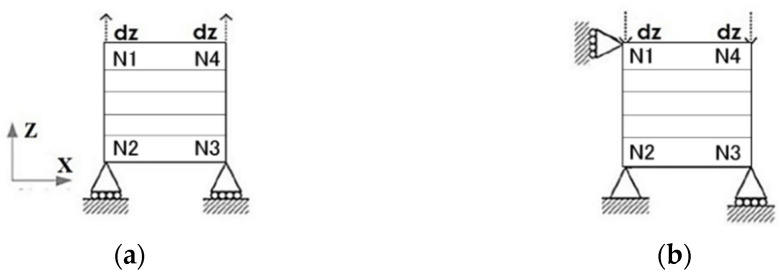
Tension load (**a**) and compression load (**b**).

**Figure 7:**
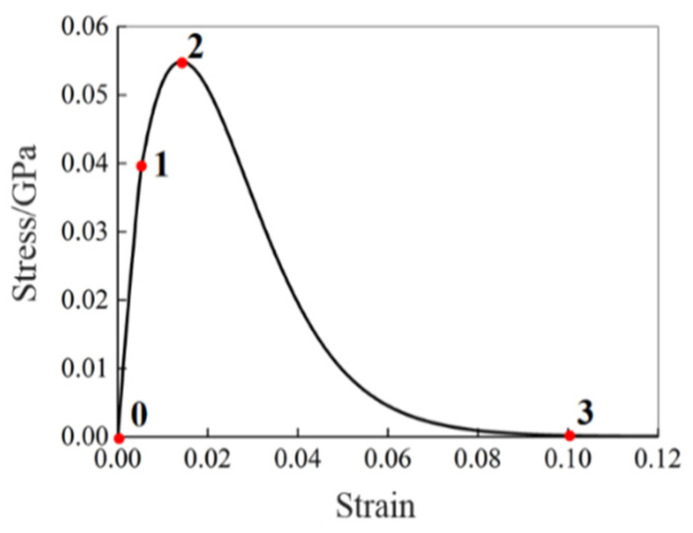
Typical stress–strain curve of the [90] single-element model, point 0 represents the initial state of the material before any load is applied, point 1 is in the early stages of the loading process where the material exhibits linear elastic behavior, point 2 represents the peak stress, also known as the ultimate strength of the material, and point 3 indicates the failure of the material.

The tension/compression load and boundary conditions are shown in Figure 6, and the stress–strain curve can be extracted. For the [90] single-element model, the typical stress–strain curve, shown in Figure 7, can be divided into the following two stages: the elastic stage (0–2) and the degradation and element deleting stage (2–3), which can demonstrate the stress–strain properties of the matrix’s elastic to plasticity.

**Figure 8:**
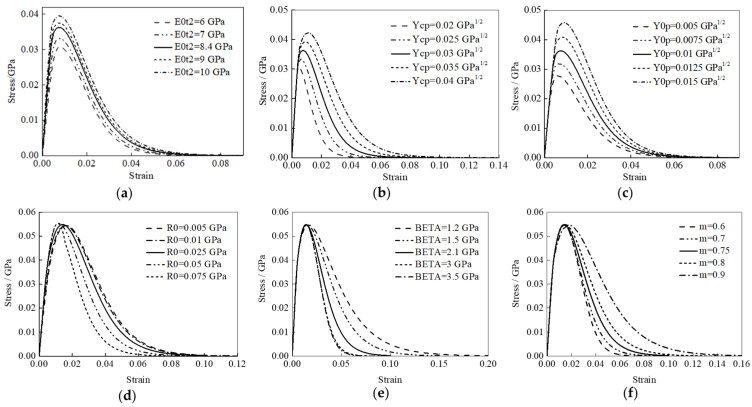
Stress–strain curves of the [90] single-element model, E0t2 (**a**), Ycp (**b**), Y0p (**c**), R0 (**d**), BETA (**e**), and m (**f**).

The E0t2 can determine the slope of the linear elastic stage in the stress–strain curves, as shown in Figure 8a. The larger the E0t2, the larger the slope of the linear elastic stage. At the same time, the E0t2 can affect the variation in the tensile stress–strain curve of the matrix from the elastic stage to the failure stage to a certain extent.

The Ycp and Y0p mainly affect the degradation stage in the stress–strain curve, as shown in Figure 8b,c, and both Ycp and Y0p can affect the tensile strength of the matrix. The larger the Ycp and Y0p, the greater the tensile strength of the matrix.

The effects of R0, BETA, and m on the stress–strain curves are shown in Figure 8d–f. By changing the values of the parameters, the downward trend of the degradation stage in the stress–strain curve can be found, and the failure strain, which causes the element deletion, is changed to varying degrees.

#### 3.3.3. Material Model Parameters

The parameters’ physical meanings and the numerical meanings of the Lavadèze material constitutive model, the Puck IFF failure criteria, and the Yamada–Sun fiber failure criteria were identified through the parametric analysis of a single-element model. Finally, the parameters are presented in Table 2.

### 3.4. Stacked Shell Model

**Figure 9:**
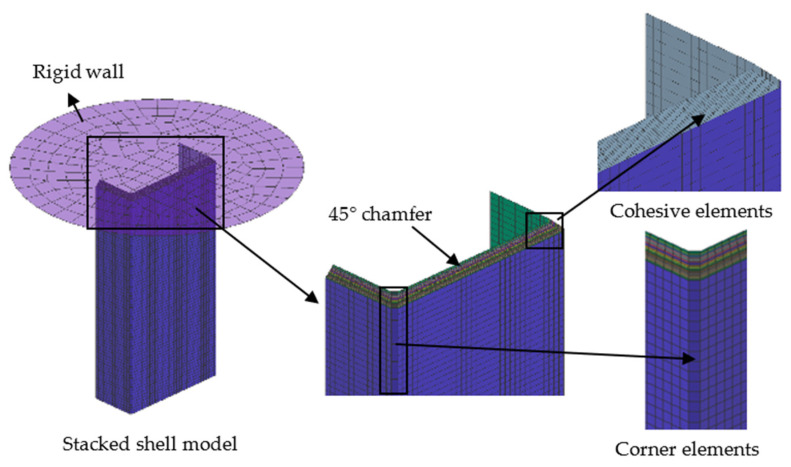
Stacked shell model of C-channels.

Based on the actual dimensions of the composite C-channels, a stacked shell FE model was developed in PAM-CRASH (2G version VPS 2020), i.e., each ply was represented by individual shell elements and stacked together using the cohesive elements to form the entire composite laminate, as shown in Figure 9. Due to stress concentration at the interface between the web and the flanges, the corner region of the C-channel was modeled by introducing a column of corner elements. The 45° outer chamfer section was created by employing a height-by-layer decrement approach. The mesh size of the web and flanges in the model was approximately 1.2 mm × 1.4 mm, while the mesh size of the corner elements was approximately 1.4 mm × 1.4 mm. This mesh density can ensure the accuracy of simulation results without significantly increasing the calculation time. The Belytschko–Tsay shell element was used, and each layer contained 4608 shell elements. The rigid wall had no deformation during the crushing process and provided a compulsive uniform velocity. Therefore, the element size, which was set to 10 mm × 10 mm, was relatively large and comprised a total of 219 elements.

To accurately simulate interlayer failure and ensure the reliability of the finite-element model, the cohesive elements were used to connect each layer, which can effectively simulate the load transmission of interlamination and delamination failure. The parameters of the cohesive element are shown in Table 3.

$G_{u}^{Ι}$ and $G_{u}^{Ⅱ}$ represent Type I and Type II fracture energy, which can be measured by a double cantilever beam test. These two parameters determine the interlaminar strength. In the model, the Pickett criteria [49] was used to couple the Type I and Type II cracks in a linear superposition, and the criteria calculation is shown in Equation (7).

(7) $$\frac{G_{i}^{I}}{G_{u}^{I}}+\frac{G_{i}^{II}}{G_{u}^{II}}=1$$

**Figure 10:**
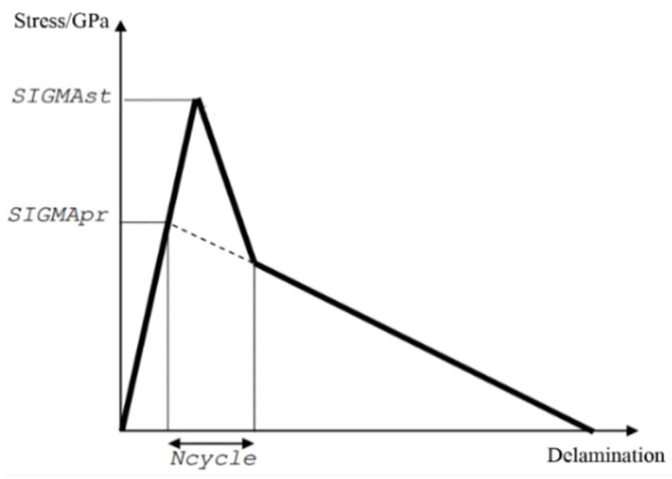
Stress-delamination propagation of cohesive element.

$G_{i}^{Ι}$ and $G_{i}^{Ⅱ}$ represent Type I and Type II crack propagation energy. In addition, the initial delamination stress, the continuous delamination stress and the stress reduction cycle number were utilized to regulate the relationship between the interlaminar crack length and the interlaminar stress. Taking the Type I crack as an example, the stress–crack length curve is shown in Figure 10.

To prevent the interlayer penetration, which can lead to inaccuracies in the simulation results, the self-contact method was employed for each layer. Accordingly, the node-to-surface contact method was used to avoid penetration between the rigid wall and the composite C-channel. For the finite-element model, the C-channel was immobilized by constraining all degrees of freedom of the bottom row of nodes situated opposite the 45° outer chamfer, while the remaining nodes were entirely free.

The strain rate effect was not considered in the finite-element models. Therefore, to enhance computational efficiency while maintaining simulation accuracy, a loading speed of 6000 mm/s was selected. Accordingly, the crushing distance was set to 44 mm, which was consistent with the test conditions. It took approximately 2–3 h to complete the simulation using a dual-core X5675 CPU and a workstation operating at a speed of 3.06 GHz.

## 4. Results and Discussion

### 4.1. Failure Morphology and Mechanism Analysis

**Figure 11:**
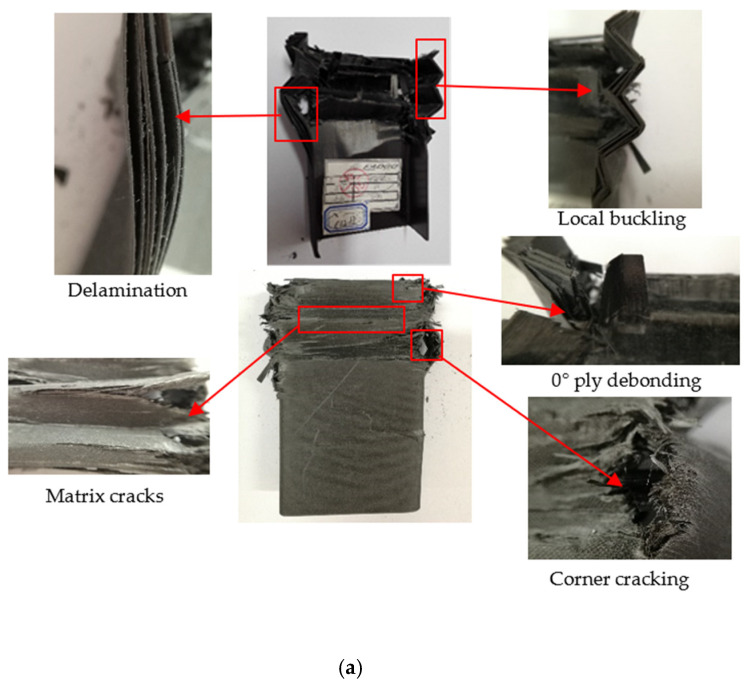

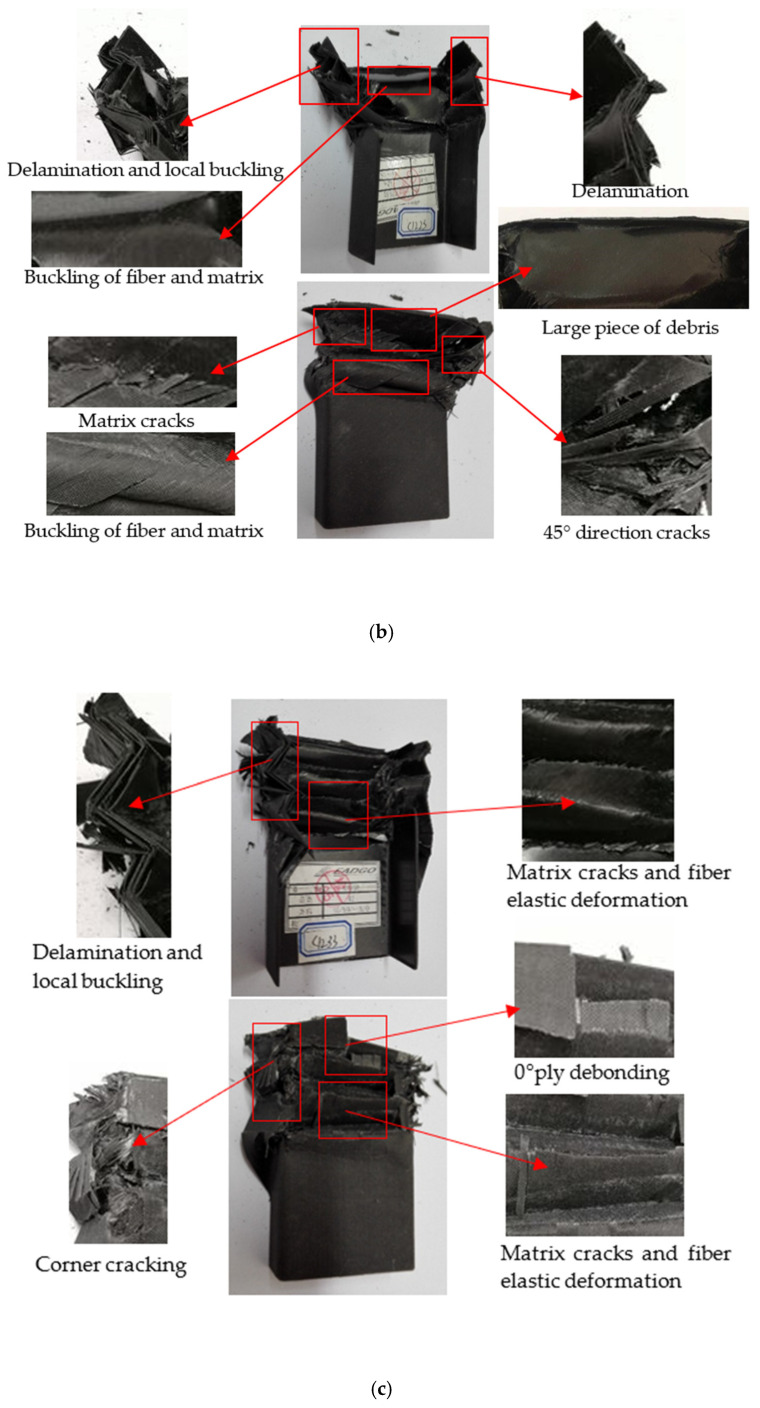
Failure morphology of the [0/90]_3s_ C-channel (**a**), [45/-45]_3s_ C-channel (**b**), and [45/90/-45/0]_3_ C-channel (**c**).

**Figure 12:**
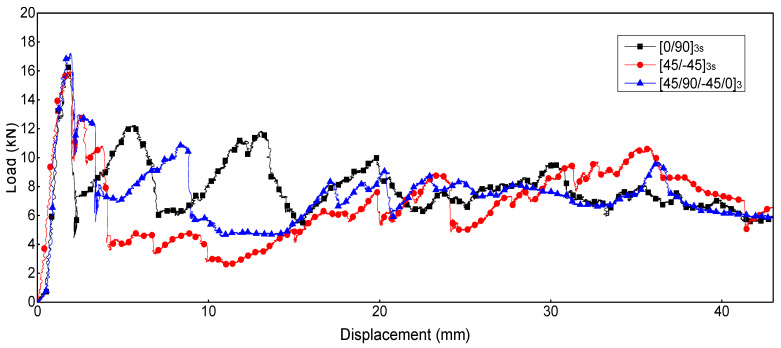
Load-displacement curves: [0/90]_3s_, [45/-45]_3s_, and [45/90/-45/0]_3_ C-channels.

The C-channels experienced a progressive crushing process under low-speed axial compression. The failure morphology of the [0/90]_3s_ C-channel, depicted in Figure 11a, shows repeated folding and collapse, leading to large intervals in the load–displacement curve (Figure 12). Horizontal cracks appeared in the flat segment and flange, with some 0° ply detaching within the flat segment. Delamination occurred at the top side of the C-channel and both flange sides. Stress concentration in the corner caused complete fiber and matrix breakage, as 0° fibers failed under axial compression, leaving the 90° fibers unable to provide support.

For the [45/-45]_3s_ C-channel, shown in Figure 11b, no folding occurred due to ±45° fiber reinforcement, which provided shear force resistance. This C-channel displayed significant elastic deformation without buckling failure or fiber fracture. However, local buckling in the flat segment caused matrix slippage along the fiber direction and numerous 45° cracks. The flange exhibited 45° matrix cracks and inward bending in the folding zone. The [45/-45]_3s_ C-channel’s fibers withstood shearing forces, preventing cracking in the corner area, but 45° direction cracks led to fiber and matrix yielding, causing the flat segment to bend outward and causing slight twisting of the C-channel. 

The [45/90/-45/0]_3_ C-channel, illustrated in Figure 11c, exhibited more compact folding. Fiber and matrix fractures, along with delamination, were observed on the top and flange sides. There was 0° layer debonding on the outside of the flat segment, and cracking occurred in the corner area. This C-channel also showed significant elastic deformation due to matrix cracks and fiber in the flat segment region.

**Figure 13:**
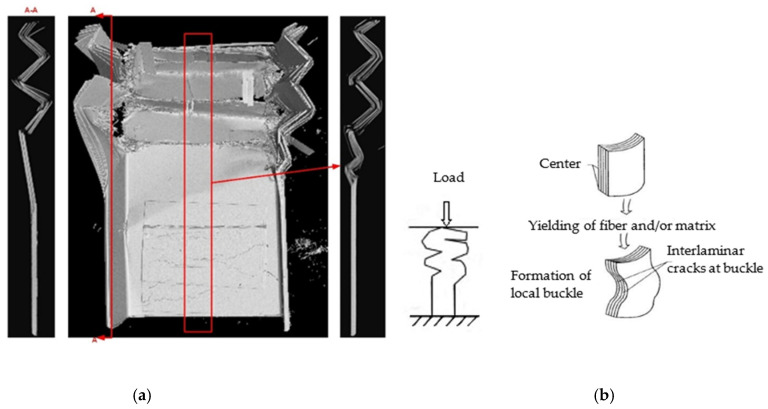
Three-dimensional image of the [0/90]_3s_ C-channel (**a**) and the local buckling failure mode (**b**).

The thin-walled composite C-channels were scanned by using CT tomography measuring machine. The three-dimensional image of [0/90]_3s_ C-channel is shown in Figure 13a. There are multiple buckling zones in the region of the flat segment and flange, many fibers and matrix are buckling without breaking, and a small amount of fiber and matrix is fracturing. The failure mode is a local buckling failure mode, resulting in superposition, as shown in Figure 13b. In the initial section, delamination occurs in the chamfer, and it is gradually crushed. Under the combination of debris and the indenter, local buckling failure occur, with fibers breaking or deforming elastically. The interlaminar shear stress are increased because of the buckling failure of fibers, and interlaminar cracks are formed, which causes the delamination and local brittle fracture of the matrix in the buckling zone. The failure mechanisms are intralaminar and interlaminar delamination, the elastic deformation of the fibers, the buckling of the fibers and matrix, the debonding and fracture of the fibers, the deformation cracking of the matrix, and corner cracking.

### 4.2. Energy-Absorbing Characteristics Analysis

Figure 12 shows the load–displacement curves of thin-walled composite C-channels under low-speed axial compression tests. For three samples with the same layer numbers and layups, the load–displacement curves are very similar, so only one of the three curves is selected to analyze the energy-absorbing characteristics. The energy-absorbing metrics are listed in Table 4.

For the thin-walled composite C-channels with different layups, the crushing loads increase approximately linearly from zero to *F*_max_: 16.294 kN for the [0/90]_3s_ C-channel, 16.006 kN for the [45/-45]_3s_ C-channel, and 17.225 kN for the [45/90/-45/0]_3_ C-channel. The chamfers of composite C-channels have been fully crushed, and the crushing loads suddenly drop, reaching a lower sustained crushing load for the remainder of crushing displacement. The [45/-45]_3s_ C-channel experiences the largest load drop, and the *F*_mean_ is the minimum. The [0/90]_3s_ C-channel and [45/90/-45/0]_3_ C-channel experience load drops less severe than exhibited by the [45/-45]_3s_ C-channel. The *F*_mean_ of the [0/90]_3s_ C-channel is the maximum, so the SEA value is also the maximum, which are 14.93% and 6.30% higher than the [45/-45]_3s_ C-channel and [45/90/-45/0]_3_ C-channel, respectively.

### 4.3. Simulation and Model Verification

**Figure 14:**
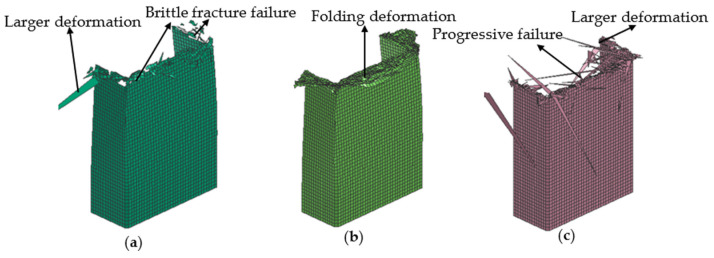
Simulation failure morphologies, 0° layer (**a**), 90° layer (**b**), and 45° layer (**c**).

The simulated failure morphologies of the 0° layer, 90° layer, and 45° layer are shown in Figure 14. During the process of the axial compression test, the 0° layer was mainly subjected to an axial compressive load, which can provide the axial stiffness for thin-walled composite C-channels. The circumferential strength and stiffness were weak, which resulted in axial bending and circumferential cracking. During the process of the axial compression simulation, brittle fracture failure mainly occurs for the 0° layer. Most of the elements were deleted after reaching the failure strain. A small part of the elements, which were not deleted, were separated from the whole structure, and a few of the elements had larger deformations, as shown in Figure 14a. 

During the axial compression test, the 90° layer was primarily supported by the matrix, leading to numerous cracks forming along the fiber direction, ultimately resulting in failure. During the process of the axial compression test, the 90° layer was primarily supported by the matrix, resulting in numerous cracks developing along the direction of the fibers, ultimately leading to failure. During the process of the axial compression simulation, the thin walls folded outward or inward, subsequently resulting in damage in the form of progressive collapse, as shown in Figure 14b. 

During the process of the axial compression test, the 45° layer was mainly subjected to shear load, transverse shear load, and adjacent interlaminar forces caused by the axial compressive loads. During the process of the axial compression simulation, a large number of elements were deleted after reaching the failure strain, demonstrating the progressive failure behavior, and some elements exhibited larger deformations, as shown in Figure 14c.

**Figure 15:**
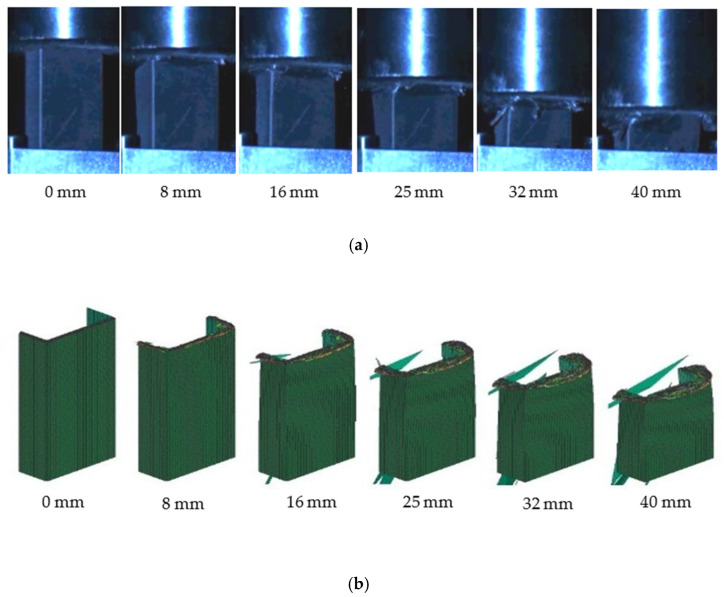
Test crushing process (**a**) and simulation crushing process (**b**).

The crushing process of the [0/90]_3s_ C-channel between the test and simulation is shown in Figure 15. With the axial movement of the steel indenter, the [0/90]_3s_ C-channel was continuously folded and exhibited progressive compression failure, resulting in a plurality of transverse cracks, and the fiber and the matrix at the corner area completely broke. The simulated progressive compression failure process is in good agreement with the test results.

**Figure 16:**
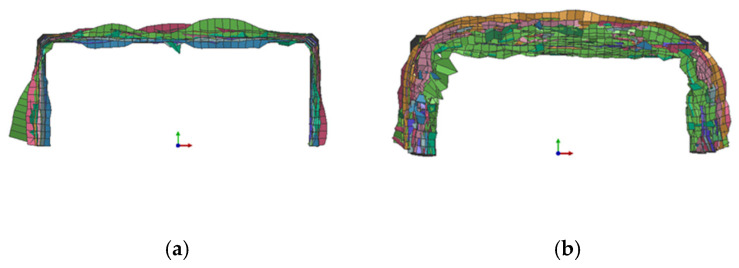
Delamination (**a**) and folding deformation (**b**).

For the stacked shell models of composite C-channels, the outer chamfer is firstly crushed under the action of the rigid wall, and delamination can be observed, as shown in Figure 16a. With the progressive crushing process, a small number of elements fail to be deleted, and many elements are deformed and stacked, as shown in Figure 16b.

**Figure 17:**
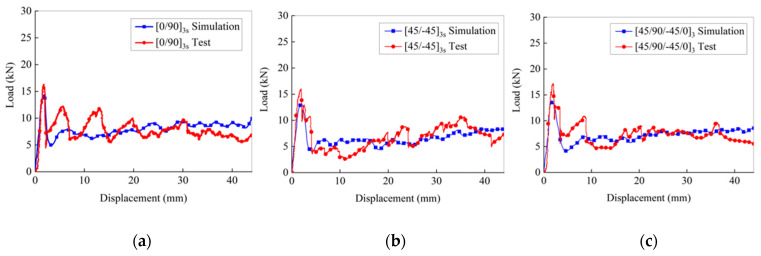
Comparison of load–displacement curves between test and simulation: [0/90]_3s_ (**a**), [45/-45]_3s_ (**b**), and [45/90/-45/0]_3_ (**c**).

A comparison of the load–displacement curves of C-channels with three different layers subject to low-speed axial compression was obtained. A low-pass digital filter (SAE 1000 Hz) was applied to filter the simulation results during post-processing. The simulated load–displacement curves aligned well with the test load–displacement curves, as shown in Figure 17. In the early stage of the axial compression process, the load increases linearly until the initial peak is reached. Subsequently, the bearing capacity of the C-channel decreases, and the load value of the load–displacement curve correspondingly decreases. Then, the compression load fluctuates up and down within a certain range.

**Figure 18:**
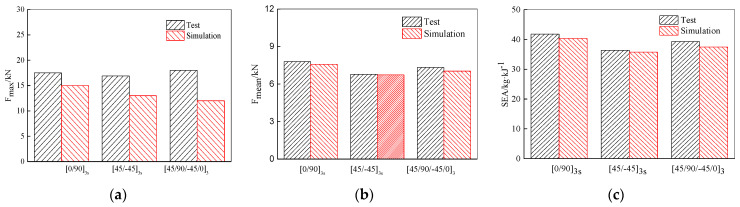
Comparison of energy-absorbing characteristics, *F*_max_ (**a**), *F*_mean_ (**b**), and *SEA* (**c**).

A comparison of the energy-absorbing characteristics of the C-channels from the test and simulation is shown in Figure 18. The simulation Fmax values are lower by approximately 5% to 30% than the test results, as shown in Figure 18a. This discrepancy is likely due to the exclusion of the strain rate effect in the material constitutive model. Additionally, material processing defects and the manufacturing process contribute to significant dispersion in the Fmax values. The failure behavior of the corner area is complex, and the finite-element model, while simplified, further complicates the accurate simulation of Fmax. However, the differences in *F*_mean_ and *SEA* between the simulation and test results are within 6%, as shown in Figure 18b,c. The model can effectively calculate the energy-absorbing characteristics of specimens and reflect their failure modes.

## 5. Conclusions

In this paper, low-speed axial compression tests were conducted on carbon fiber T700/MTM28 composite C-channels with different layups. Load–displacement curves and failure damage modes were obtained. Based on the parametric analysis for the [0] and [90] single-element models, some key parameters were determined and stacked shell models of thin-walled composite C-channels were established. The failure mechanism and energy-absorbing characteristics were analyzed, and the reliability of the stacked shell model was verified against the test results. Some conclusions were drawn as follows:

(1) The thin-walled composite C-channels with [0/90]_3s_, [45/-45]_3s_, and [45/90/-45/0]_3_ exhibit a stable progressive crushing progress characterized by local buckling. This stability is crucial for energy-absorption applications in crashworthiness.

(2) The primary failure mechanisms identified include intralaminar and interlaminar delamination, elastic deformation of fibers, buckling of fiber and matrix, debonding and fracture of fibers, deformation cracking of matrix, and corner cracking. These failure modes interact to dissipate energy during compression, enhancing the energy-absorption capacity of the composite structures.

(3) The physical and numerical meanings of the Lavadèze single-layer model, the Puck IFF failure criteria, and the Yamada–Sun fiber failure criteria are clarified by studying the stress–strain relationship of the single-element model. The proposed values of the material model are given by parametric analysis.

(4) The specific energy absorption (SEA) values for the different layups are 18.5kJ/kg([0/90]3s), 15.2kJ/kg([45/-45]3s), and 17.1kJ/kg([45/90/-45/0]3). The mean crushing force (Fmean) values are 14.5kN([0/90]3s), 12.8kN([45/-45]3s), and 13.9kN([45/90/-45/0]3). The stacked shell model can accurately capture the interlayer matrix failure and interface delamination cracking behavior observed in the experiments. The simulations exhibit a discrepancy of less than 6% when compared to the experimental results, indicating a strong correlation between the two sets of data. This modeling approach provides a reliable tool for predicting the energy-absorption characteristics of composite structures.

(5) The methods used in this study, including parametric analysis and finite-element modeling, offer robust tools to assess the service performance of materials produced on a large scale for high-performance applications.

The results obtained in this study demonstrate that the molecular-level interactions and matrix–fiber interactions are fundamental in determining the mechanical properties and failure modes of composite materials. A thorough understanding of these interactions enables more accurate predictions of composite behavior under load and facilitates the design of composites with optimized performance characteristics. Our models, which incorporate these molecular-level considerations, provide a robust framework for analyzing and predicting the energy absorption and failure mechanisms of composite structures.

## Figures and Tables

**Table 1:** Summary of the parametric studies performed.

Parameter	Physical Significance	Unit	Parametric Variation			
E0t1	Young’s modulus in Direction 1	GPa	50	100	200	300
EPSIfti	Tensile fiber initial strain	-	0.005	0.01	0.018	0.02
EPSIftu	Tensile fiber ultimate strain	-	0.018	0.02	0.03	0.035
Dftu	Tensile fiber ultimate damage	-	0.2	0.4	0.8	1
Xt11	Tensile fiber strength/strain for failure	-	0.05	0.1	0.3	0.4
E0c1	Compressive fiber Young’s modulus in Direction 1	GPa	50	100	200	300
GAMMA	Compressive factor of modulus correction	-	0	0.2	0.6	0.8
Dfcu	Compressive fiber ultimate damage	-	0.2	0.4	0.8	1
Xc11	Compressive tensile fiber strength/strain for failure	-	0.05	0.08	0.2	0.3
E0t2	Young’s modulus in Direction 2	GPa	6	7	9	10
Ycp	Critical transverse damage limit	GPa^1/2^	0.02	0.025	0.035	0.04
Y0p	Initial transverse damage limit	GPa^1/2^	0.005	0.0075	0.0125	0.015
R0	Initial yield stress	GPa	0.005	0.01	0.05	0.075
BETA	Hardening law multiplier	GPa	1.2	1.5	3	3.5
m	Hardening law exponent		0.6	0.7	0.8	0.9
DAMpost	Post damage of matrix	-		1	0.9	0.85

**Table 2:** Material model parameters.

Parameters	Definition	Unit	Value	Measurement
RHO	Mass density of ply material	g/cm^3^	1.52	ASTM D792 [56]
E0t1	Young’s modulus in Direction 1 of fiber	GPa	126	ASTM D3039 [57]
E0t2	Young’s modulus in Direction 2 of fiber	GPa	8.40	ASTM D3039
E0c1	Compressive Young’s modulus of fiber	GPa	116	ASTM D3410 [58]
G012	Shear modulus of 1,2-plane	GPa	3.69	ASTM D3518 [59]
NU12	Poisson’s ratio in 1,2-plane	-	0.3	ASTM D3039
R22+	Matrix tensile transverse strength	GPa	0.0554	ASTM D638 [60]
R22-	Matrix compressive transverse strength	GPa	0.225	ASTM D695 [61]
R12	Matrix shear strength	GPa	0.2	ASTM D5379 [62]
EPSIfti	Tensile fiber initial strain	-	0.0217	
Ycp	Critical transverse damage limit	GPa^1/2^	0.0596	Calibrated by material coupon level test results.
Y0p	Initial transverse damage limit	GPa^1/2^	0.01	
Dmax	Maximum allowed damage value for shear damage and transverse damage	-	1	
GAMMA	Compressive factor of modulus correction	-	0	
R0	Initial yield stress	GPa	0.025	
BETA	Hardening law multiple	GPa	2.1	
m	Hardening law exponent	-	0.75	
Dpost	Post damage of matrix	-	0.96	
Dftu	Tensile fiber ultimate damage		0.7~0.85	
Dfcu	Compressive fiber ultimate damage		0.7~0.85	
EPSIftu	Tensile fiber ultimate strain	-	0.025	
Xt11	Tensile fiber strain for failure	-	0.52	
Xc11	Compressive tensile fiber strain for failure	-	0.38	

**Table 3:** Cohesive element parameters.

Parameters	Definition	Unit	Values
hcont	Distance for kinematic computation	mm	0.3
E0	Normal modulus	GPa	4
G0	Shear modulus	GPa	2.5
SIGprpg	Normal stress to continue delamination	GPa	0.098
GAMAprpg	Shear stress to continue delamination	GPa	0.094
$G_{u}^{Ι}$	Mode I fracture energy	J/mm^2^	0.00047
$G_{u}^{Ⅱ}$	Mode II fracture energy	J/mm^2^	0.002
SIGstrt	Normal stress to initiate delamination	GPa	0.1
GAMAstrt	Shear stress to initiate delamination	GPa	0.1
Ncycle	Stress reduction cycle number		100

**Table 4:** Energy-absorbing metrics of C-channels.

Layups	*F*_max_/kN	*F*_mean_/kN	*EA*/kJ	*SEA*/(kJ/kg)
[0/90]_3s_	16.294	7.801	345.058	41.793
[45/-45]_3s_	16.006	6.776	300.236	36.364
[45/90/-45/0]_3_	17.225	7.329	324.625	39.318

## Data Availability

The original contributions presented in the study are included in the article, further inquiries can be directed to the corresponding author.
